# Changes in well-being during the COVID pandemic: A longitudinal study of older adults

**DOI:** 10.1093/geroni/igab046.2735

**Published:** 2021-12-17

**Authors:** Andrea Huseth-Zosel, Heather Fuller

**Affiliations:** 1 North Dakota State University, Fargo, North Dakota, United States; 2 North Dakota State university, Fargo, North Dakota, United States

## Abstract

The COVID-19 pandemic is a public health crisis the world has not seen in a century, with older adults faced with unique impacts due to their increased vulnerability and need to social distance. This research examines changes in physical and mental health and quality of life among older adults in the upper Midwest during the COVID-19 pandemic. Seventy older adults aged 70-97 participated in three phone interviews (April [Time 1], June [Time 2], and October [Time 3] 2020) focusing on experiences coping with the pandemic and understanding overall changes in well-being. Participants rated their quality of life, physical health, and mental health on a scale from 1 to 5 with 1 being “Poor” and 5 being “Excellent.” Self-reported quality of life, mental health, and physical health initially declined between retrospective pre-COVID and Time 1 scores, with gradual increases seen across all three variables for Time 2 and Time 3 scores. Thematic analysis of qualitative responses for each interview wave identified salient themes of: 1) reduced quality of life, 2) distraction and routine, 3) loss and uncertainty, and 4) resilience and adaptation. The significance and meaning of these themes shifted across each time point. For example, the reduced quality of life theme initially encompassed loss of activities, later shifted to concerns about struggles to maintain relationships, and finally focused on hope for the future. Findings will be discussed in light of the significance of change over time as well as policy and practice implications for older adults.

